# A Case and Review of Ophthalmomyiasis Caused by *Oestrus ovis* in the Central Valley of California, United States

**DOI:** 10.1177/2324709619835852

**Published:** 2019-04-04

**Authors:** Carlos D’Assumpcao, Addie Bugas, Arash Heidari, Sandra Sofinski, Rick A. McPheeters

**Affiliations:** 1 Kern Medical – UCLA, Bakersfield, CA, USA; 2 Ross University School of Medicine, Miramar, FL, USA

**Keywords:** *Oestrus ovis*, ophthalmomyiasis externa, sheep bot fly

## Abstract

Ophthalmomyiasis externa is the infestation of external ocular structures most commonly by *Oestrus ovis*, sheep nasal bot fly, which have a pupal stage in soil. Farmers and shepherds are commonly affected but rarely in urban areas. This is the first case of *Oestrus ovis* ophthalmomyiasis externa in California since Catalina Island 1986. No livestock exposure was noted. Manure fertilizer sourced from grazing fields of natural hosts was used on a local urban sports field and is the suspected source.

## Introduction

Myiasis is a zoonotic infestation with dipterous larvae. Ophthalmomyiasis is the infestation of ocular or orbital tissues by these dipterous larvae.^1,2^ The most common presentation of ocular myiasis is ophthalmomyiasis externa, in which infestation of dipterous larvae occurs in the superficial external ocular structures.^3-5^ The most common cause of ophthalmomyiasis in humans is *Oestrus ovis*, which usually affects shepherds and farmers due to proximity to natural hosts,^2-4^ such as sheep,^6^ llamas,^7^ and goats.^8^

Lifecycle begins as eggs are fertilized and hatched within the body of a female *O ovis*. Live larvae, 1 mm in length, are excreted into mucus drops on the exterior of the maternal fly. Larvae are deposited into the moist mucus membranes of principle hosts. First instar larvae travel up into nasal sinuses of their host and continue their maturation to the third instar stage over 2 to 10 months. Third instar larvae drop from nasal cavity to the ground for pupation in a chrysalis. Adult lifespan is 2 to 4 weeks. Total lifespan is largely dependent on temperature and environmental conditions.^9^

Infection of accidental human hosts is hypothesized to occur by inoculation of live larvae in a mucus drop onto moist membranes by a female *O ovis*. Ocular involvement is most common, followed by nasal and gastrointestinal mucus membranes. Ophthalmomyiasis interna due to *O* ovis is very rare^10,11^ or may be underreported due to difficulty isolating larvae from the vitreous humor for identification.

Review of literature revealed case reports of ophthalmomyiasis externa by *O ovis* in the United States most recently in Hawaii^12,13^ and Dallas, Texas.^14^ The largest reported single location of ophthalmomyiasis by *O ovis* in the contiguous United States is Catalina Island, California.^3,8,15,16^

Presented here is the first documented case of ophthalmomyiasis externa by *O ovis* in the Central Valley of California.

## Case Report

A 16-year-old male with past medical history of recurrent sinusitis and otitis media requiring bilateral tympanostomy tube placement and tonsillectomy at age 4 and environmental allergies requiring allergen immunotherapy presents with 1-day history of irritation, redness, and mobile swimming foreign body sensation in left eye.

**Figure 1.**
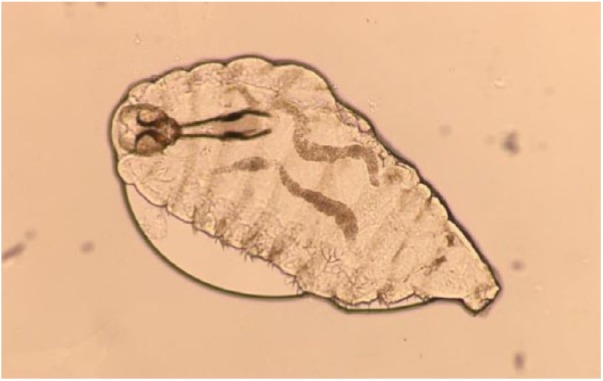
*Oestrus ovis*, first instar larvae, saline wet mount, light microscopy, 10× magnification.

**Figure 2.**
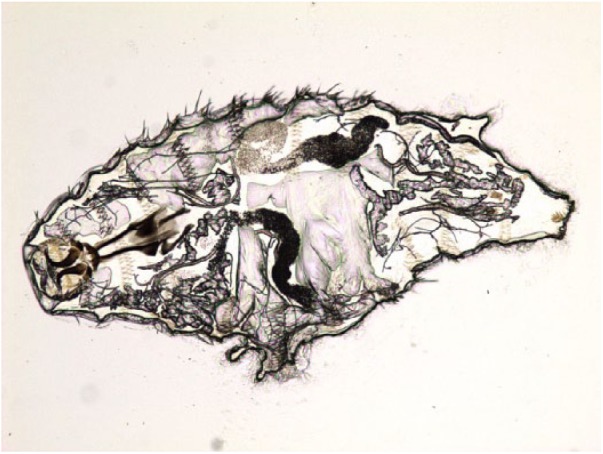
*Oestrus ovis*, first instar larvae, saline wet mount, light microscopy but with phase contrast, 10× magnification.

**Figure 3.**
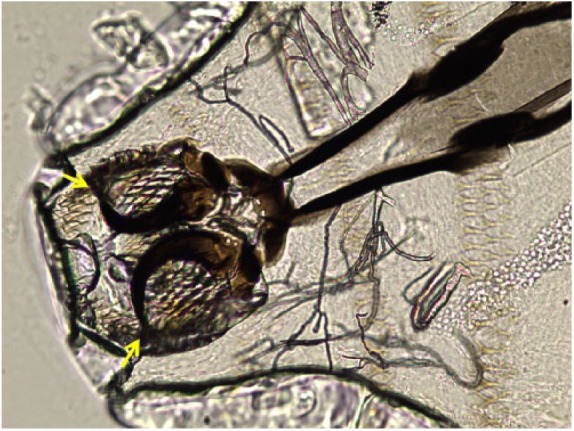
*Oestrus ovis*, first instar larvae, detail of cephalopharyngeal skeleton with anterior hooks (arrows), saline wet mount, light microscopy, phase contrast, 40× magnification.

**Figure 4.**
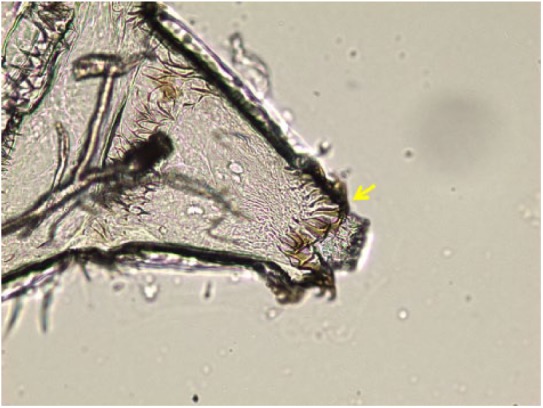
*Oestrus ovis*, first instar larvae, detail of caudal spines (arrow), saline wet mount, light microscopy, phase contrast, 40× magnification.

At onset of symptoms, the patient was participating in after-school sports practice on a natural grass football field at a local high school in a southern San Joaquin Valley municipality in California. Review of field maintenance records reveal recent fertilization with manure. The patient’s eye became increasingly red and irritated, which was not relieved with artificial tears or irrigation with tap water. Mechanical removal was successfully completed by the patient and in the emergency department. Eleven larvae removed were consistent with the first instar stage of *O ovis* (Figures 1-4) as per pathological examination on saline wet mount with light microscopy and comparison with standard entomologic texts. The patient had left conjunctiva injection, intact extra ocular movements, and bilateral normal visual acuity. On slit lamp examination, multiple larvae were appreciated in the bulbar conjunctiva and palpebral fornix of left eye. They did not appear to be burrowing. The patient was given polysporin-bacitracin-neomycin ophthalmic solution and albendazole 400 mg daily for 3 days for prophylaxis. Follow-up ophthalmology examination confirmed clearance of larvae at 2 days.

Family history was significant for diabetes, hypertension, and dyslipidemia. Travel history was limited to southern San Joaquin Valley, California. Animal exposure includes 4 dogs and 1 young chicken at home. Denies other livestock exposure in his neighborhood, specifically no exposure to sheep, goats, bovines, or other livestock.

## Discussion

This is the first reported case of ophthalmomyiasis externa in California since Catalina Island in 1986 (see Table 1). Catalina Island is home to wild goats.^8^ Those that developed ophthalmomyiasis externa were residents or visitors to the island.^3,8,15,16^ Our patient’s presentation was similar to these examples of reported ophthalmomyiasis externa cases.^17,18^ No systemic treatment for this condition exists, only mechanical removal. Antibiotics can prevent bacterial co-infection.^16^

**Table 1.** Human Cases of Ophthalmomyiasis Externa Caused by *Oestrus ovis* in the United States as Reported in the Literature.

Reference/Year	Age (Years)	Sex	State/Location	Exposure	Larval and Stage
Current case report	16	Male	Southern San Joaquin Valley, California	Sports practice on natural grass football field	First instar
Kajioka et al^12^ (2004)	16	Male	Hawaii	Unloading a Christmas tree	First instar
Sigauke et al^14^ (2003)	16	Female	Dallas, Texas	Suspected eggs on hand	4 mm × 1 mm
Yoshimoto et al^13^ (1997)	63	Male	Oahu, Hawaii	Working on a car	First instar
Heyde et al^16^ (1986)	28	Male	Los Angeles, California	Sightseeing and snorkeling at Catalina Island	First instar
Reingold et al^8^ (1984)	17	Female	Catalina Island, California	Vacation to Catalina Island	First instar
Prior to 1981, according to Reingold et al^8^	5 to 70	7 females and 8 males	Catalina Island, California	Catalina Island	Unknown

It is not known if the patient’s history of environmental allergies increased his risk of larvae acquisition. He had no complaints of allergic conjunctivitis while playing football. As well, his unilateral conjunctivitis was more akin to reaction to foreign body rather than to foreign antigen. His propensity to environmental allergens did not seem to increase his reaction to foreign larvae out of proportion compared with published case reports.

In review of past cases in the United States, the unique feature unifying of all the cases was being outside participating in outdoor activities. Teenagers were more affected, likely due to their propensity to be outside, but seniors over age of 65 years were also affected, if they were also outside at the time. Environmental allergies were not noted in the published case reports.

Manual removal is primary therapy. Irrigation with a suitable solution or with jeweler’s forceps, or a lightly moistened small cotton bud have been suggested.^13^ To prevent larvae from burrowing deeper into ocular tissues, ocular paralytics have been suggested in the literature. Cocaine 4% to 5% solutions, lidocaine, or pilocarpine 1% to 4% ophthalmic solutions have been reportedly effective.^13,16^ Paraffin oil has been suggested to deprive oxygen from the larvae.^14^ Proparacaine hydrochloride 0.5% ophthalmic solution did not appreciably slow larval movements,^13^ but was successfully used in the past^8^ to assist with removal. However, despite paralysis, larvae may cling to tissue with their hooks. Neomycin, bacitracin, and polymyxin B ophthalmic ointment have also been used successfully to presumably suffocate the larvae, facilitating their later removal at follow-up.^8,16^ With higher larval loads, multiple sessions may be required.^19^ Our patient was successfully treated with self-irrigation with water and small cotton buds at home and the emergency room without need for paralysis.

Post removal therapy revolve around prevention of superimposed bacterial infection and reduction of any inflammation that may damage ocular structures. Neomycin, bacitracin, and polymyxin B ophthalmic ointment,^8,16^ and hydrocortisone^8^ have been used. No reported superimposed bacterial infections have yet been reported in patients who did not receive antibiotics after manual removal. However, given the source of the larvae, patients may prefer empirical prophylaxis to prevent bacterial infection. Of note, our patient did develop ocular irritation at follow-up likely from the ophthalmic ointment prescribed, which then resolved after ointment was no longer used. Formal ophthalmologist evaluation for possible complication of ophthalmomyiasis interna is strongly suggested.

While no reported cases used albendazole or ivermectin systemic therapy, our physicians erred on the side of caution at time of discharge from our emergency department given the lack of larval identification at disposition and physician exposure to ophthalmomyiasis externa by *O ovis* within the geography of our medical care, hence the impetus for this case report.

Natural occurrence of *O ovis* in sheep from New Mexico, western Texas, southern Colorado, and eastern Arizona was studied in 1962.^6^ Overall, 91% of 720 sheep over a 28-year period (1934-1961) were found to be infected. Peak infection was between October and December.

Increase in the population of livestock that are natural hosts of *O ovis* near urban areas may increase incidence of ophthalmomyiasis externa in accidental human hosts. In 2013 in Marseille, France, a 3-week festival involving 3000 sheep and goats and greater than 300 000 spectators resulted in 5 cases of ophthalmomyiasis by *O ovis* in a 4-week period in an area with a previous incidence of one case in 5 years.^20^

As for California, Southern San Joaquin Valley has been historically a diasporic home to Basque shepherds from the autonomous community of Basque Country in northern Spain since the 1970s due to immigration.^21^ This shepherding community has been dwindling in number in recent due to environmental and socioeconomic factors.^22^

Fertilizer sourced from livestock grazing pens may be a unique source of infection in areas that do not have livestock. *O ovis* lifecycle involves a pupal stage in the soil.^9^ Collecting manure from host animals to be used as fertilizer in urban sports fields brings unhatched pupas into the proximity of human hosts. This can be considered a public health risk.

## Conclusion

Although ophthalmomyiasis externa is uncommon in North America, prevalence can change with the exposure to host livestock or its manure. Early diagnosis and management of ophthalmomyiasis externa is important in preventing complications, such as ophthalmomyiasis interna,^10,11^ or auricular,^23^ nasal,^9,23,24^ pharyngeal,^23,25^ or laryngeal^23^ myiasis. There is no known systemic treatment for this condition, only mechanical removal. Antibiotics can be used to prevent bacterial co-infection. The use of manure as fertilizer on fields that people may have close interaction with should be limited, especially if the manure is sourced from grazing fields of animal hosts of *Oestrus ovis*.
